# Effect of Vestibular Rehabilitation Therapy in PPPD: Short-Term Results from a Prospective Observational Study

**DOI:** 10.3390/jcm14217761

**Published:** 2025-11-01

**Authors:** Viktoras Simanavicius, Daiva Mockeviciene, Marija Lebedeva, Rafaela Cavalheiro do Espírito Santo, Laura Zaliene, Arnas Staskevicius, Cesar Agostinis-Sobrinho

**Affiliations:** Department of Holistic Medicine and Rehabilitation, Faculty of Health Sciences, Klaipeda University, H. Manto g. 84, LT-92294 Klaipėda, Lithuania; daiva.mockeviciene@ku.lt (D.M.); rafaela.santo@ku.lt (R.C.d.E.S.); laura.zaliene@ku.lt (L.Z.); cesar.agostinis@ku.lt (C.A.-S.)

**Keywords:** persistent postural-perceptual dizziness (PPPD), vestibular rehabilitation therapy (VRT), dizziness, quality of life, functional improvement

## Abstract

**Objective:** This short-term prospective observational study aimed to evaluate the efficacy of vestibular rehabilitation therapy (VRT) in Patients Diagnosed with Persistent Postural-Perceptual Dizziness (PPPD). **Methods:** Given the exploratory design, the small sample (*n* = 25) and absence of a formal power calculation limit precision, findings should be interpreted as preliminary, and confirmatory trials are warranted. Patients were assessed before (T1), immediately after a five-week vestibular rehabilitation program (T2), and again three months later without continued therapy (T3). Clinical symptoms were assessed using the Dizziness Handicap Inventory (DHI). A Generalized Estimating Equations (GEE) model was used to analyze changes in dizziness-related physical, emotional, and functional impacts over time, accounting for sex and its interaction with time. Statistical significance was tested using the Wald test, with results reported as estimated means and standard errors (SEs), and a significance level set at *p* ≤ 0.05. **Results:** The mean age of participants was 44.48 ± 14.43 years, and the majority were women (84%). In the functional domain, the mean score difference was 6.69 points between T1 and T2 (*p* = 0.018), 7.11 points between T1 and T3 (*p* = 0.013), and 0.42 points between T2 and T3 (*p* > 0.05). In the emotional domain, the mean difference was 4.12 points between T1 and T2 (*p* = 0.008), 4.40 points between T1 and T3 (*p* = 0.005), and 0.29 points between T2 and T3 (*p* > 0.05). In the physical domain, the mean difference was 3.77 points between T1 and T2 (*p* = 0.024), 4.32 points between T1 and T3 (*p* = 0.009), and 0.55 points between T2 and T3 (*p* > 0.05). For the total score, the mean difference was 14.58 points between T1 and T2 (*p* = 0.005), 15.83 points between T1 and T3 (*p* = 0.003), and 1.25 points between T2 and T3 (*p* > 0.05). The moment variable had a statistically significant effect across all domains. Sex had a significant effect only in the emotional domain, with women consistently reporting higher scores than men. **Conclusions:** This study demonstrates that a five-week vestibular rehabilitation program significantly improves the physical, emotional, and functional impacts of dizziness in patients with PPPD, with these benefits largely sustained three months after the intervention. Emotional improvements were particularly notable among women, highlighting potential sex-related differences in response to treatment. These findings underscore the importance of addressing emotional health in PPPD management and support the long-term effectiveness of vestibular rehabilitation in improving patient outcomes.

## 1. Introduction

Persistent Postural-Perceptual Dizziness (PPPD) is a chronic disorder characterized by functional, structural, and psychological alterations [1,2]. PPPD is relatively common: in specialty dizziness/vestibular clinics it accounts for roughly 15–20% of patients with vestibular symptoms [3], with point estimates of ~19% reported in tertiary vertigo cohorts [4]; about one quarter (~25%) of patients develop PPPD after an acute or episodic vestibular disorder [3]. Population-level data are limited, but a UK primary-care analysis suggested that PPPD may affect ~4% of individuals registered with general practitioners [5]. PPPD most often presents in adults aged 30–50 years and shows a female predominance [6].

Clinical symptoms can appear alone or in combination and include dizziness, unsteadiness, or non-spinning vertigo [2]. Due to these features, patients with PPPD experience reduced quality of life and worsening mental health, such as depression and anxiety [7,8,9]. Notably, PPPD shows a significant correlation with depressive and anxiety symptoms [9].

Management remains unclear. Recent systematic reviews and meta-analyses further synthesize evidence for non-pharmacological approaches in PPPD, underscoring both the promise of VRT and the need for higher-quality trials [10,11,12]. Although a review suggested a potential effect of selective serotonin reuptake inhibitors (SSRIs) and serotonin–norepinephrine reuptake inhibitors (SNRIs) on PPPD symptoms, a recent systematic review found no randomized, placebo-controlled evidence supporting the efficacy of these pharmacological treatments [13].

Among non-pharmacological options, vestibular rehabilitation therapy (VRT) is considered promising for PPPD [10,11,12]. Conceptually, VRT targets postural control and habituation to provocative motion/visual stimuli [12] and has been reported to improve balance/sensory organization and subjective dizziness symptoms [14,15,16,17,18,19]; effects on standing-posture stability are inconsistent [20], and findings for disease-specific quality of life and mental health are mixed [10,11,12,14,15,16,17,18,19], including reports of associations with depression/anxiety [7,8,9]. Overall, the evidence specific to PPPD is still developing, with gaps in durability and comparative effectiveness.

Accordingly, this prospective observational study evaluated the impact of supervised, clinic/hospital-based VRT (with an adjunct home program) on PPPD symptoms in a single-center cohort recruited in Lithuania, focusing on changes in functional, emotional, and physical domains and the DHI total score from baseline to post-intervention and to three-month follow-up.

## 2. Materials and Methods

### 2.1. Design

This prospective observational study was conducted at a single center in Lithuania (Klaipėda University/Republican Hospital). Participants were consecutively recruited at this site. The sample was recruited by convenience and consecutively, and all participants who were included underwent the intervention. Ethical approval for the study was granted by the Kaunas Regional Biomedical Research Ethics Committee (20 May 2022, Nr. BE-2-45). The study followed the principles of the Declaration of Helsinki, and all participants provided written informed consent. The study was conducted at the Laboratory of Neurosensorimotor Diagnostics within the Department of Holistic Medicine and Rehabilitation at Klaipėda University. Participants were referred from the Republican Hospital and were diagnosed with PPPD by a neurotologist according to the Bárány Society criteria prior to enrollment. The study period was June–August 2022.

### 2.2. Participants

Inclusion criteria. (i) Age ≥ 18 years; (ii) diagnosis of PPPD established by a neurotologist according to the Bárány Society criteria (symptoms of dizziness, unsteadiness, or non-spinning vertigo present on most days for ≥3 months; exacerbation by upright posture, active or passive motion, or complex/visually rich stimuli; precipitated by a balance-disrupting event; causes distress/functional impairment; and not better explained by another disorder); and (iii) no additional vestibular or psychological interventions during the study period [2]. Exclusion criteria. (i) Severe cognitive or motor impairment precluding participation in rehabilitation; (ii) pregnancy; (iii) comorbid health conditions that contraindicated participation in rehabilitation; and (iv) alternative primary central or peripheral disorders accounting for the symptoms, ruled out after neurotological evaluation (e.g., active benign paroxysmal positional vertigo; Ménière’s disease in an active/hydropic phase; acute vestibular neuritis/labyrinthitis with ongoing deficits; superior semicircular canal dehiscence or other third-window syndromes with pathognomonic signs; vestibular schwannoma under active treatment; primary vestibular migraine at enrollment; brainstem/cerebellar stroke, demyelinating disease, cerebellar degeneration, or other structural central causes). Patients with residual vestibular deficits after a prior event were eligible only if their presentation fulfilled PPPD criteria and no active alternative disorder better explained the symptoms.

### 2.3. Neurotological Examinations and Diagnostic Workflow

All candidates underwent a standardized assessment by a neurotologist to confirm PPPD and to exclude alternative causes of chronic dizziness. The workflow included:

1. Clinical history focused on PPPD features: symptom quality and duration (≥3 months); day-to-day pattern; exacerbation by upright posture, motion, and complex/visually rich environments; precipitating vestibular or medical events; and functional impact.
2. Bedside neurological and oculomotor examination, including assessment of spontaneous and positional nystagmus, smooth pursuit and saccades, and the head-impulse test (video head-impulse when available).
3. Positional maneuvers to exclude BPPV (Dix–Hallpike and supine roll tests).
4. Audiovestibular testing as indicated: videonystagmography with caloric testing; pure-tone audiometry and tympanometry; cervical/ocular VEMPs when clinically warranted.
5. Postural/balance assessment (e.g., Romberg and tandem stance; instrumented posturography where available).
6. Screening for relevant comorbidities, including migraine and autonomic symptoms, and screening for anxiety and depressive symptoms to document associated features.
7. Neuroimaging when indicated (e.g., red flags or atypical findings) to exclude central structural causes.

Enrollment required fulfillment of all Bárány Society PPPD criteria (A–E) and the absence of a better explanatory central or peripheral diagnosis; patients meeting criteria were enrolled, and those with an alternative diagnosis were not included [2].

### 2.4. Outcome

Dizziness Handicap Inventory (DHI). Dizziness-related disability was assessed using the DHI, a 25-item questionnaire scored 0 (No), 2 (Sometimes), or 4 (Yes). The instrument yields three subscores: Physical (7 items; range 0–28), Emotional (9 items; range 0–36), and Functional (9 items; range 0–36). The total DHI is the sum of all items (range 0–100), with higher scores indicating greater handicap; commonly used severity bands are 0–30 (mild), 31–60 (moderate), and 61–100 (severe) [21].

Option B (translation used, formal validation not published): “Because a fully validated [Lithuanian] version is not available, we used a forward–back translation of the DHI following standard guidelines, with reconciliation by a bilingual neurotologist and pilot testing for clarity in five patients prior to data collection; we report total and subscale scores as described above.”

### 2.5. Intervention

Participants were assessed prior to the intervention (T1). Vestibular rehabilitation therapy (VRT) was delivered twice weekly for five weeks (10 supervised sessions). Each session lasted 30–40 min and followed a standardized structure with individual tailoring. A post-intervention assessment was conducted at T2, and a follow-up at T3 (3 months) during which no supervised VRT was provided.

Session structure and equipment. Sessions began with a brief symptom check and heart-rate/blood-pressure screening, followed by graded exercises performed in seated and then standing positions, progressing to dynamic tasks (sit-to-stand, gait). No special equipment was required; optional items included a metronome (for pacing), a foam pad (reduced-stability surface), and printed visual patterns (for optokinetic/visual-motion exposure). Exercises were performed for ~2 min per set, typically 2 sets each, with rest as needed. Difficulty was adjusted based on tolerance and performance.

Exercise components.

1. Habituation and visual-motion desensitization (core for PPPD).
  ○ Repeated exposure to provocative head/body movements (e.g., repeated sit-to-stand; head turns/nods in sitting and standing; body turns in place).
  ○ Visual-motion exposure using busy visual scenes (e.g., patterned backgrounds, scrolling text/videos on a tablet) and optokinetic stimuli; eyes-open/eyes-closed variations were used judiciously.
  ○ Parameters: continuous or interval exposure for ~2 min/set; symptom-titrated to evoke mild–moderate transient increase in symptoms that settled within ~15 min.
2. Gaze-stabilization (vestibule-ocular reflex, VOR).
  ○ VOR ×1: head turns (horizontal/vertical) while maintaining focus on a stationary target at ~1 arm’s length.
  ○ VOR ×2 (as tolerated): head and target move in opposite directions.
  ○ Progression: increase speed (guided by metronome), duration, and stance difficulty (feet together → semi-tandem → tandem; firm surface → foam).
3. Postural control and balance.
  ○ Static: Romberg, semi-tandem/tandem stance (eyes open → eyes closed), foam surface as tolerated.
  ○ Dynamic: weight shifts, step taps to marked targets, turns in place, sit-to-stand with head turns, and dual-task balance (e.g., reciting months backward while maintaining stance).
4. Gait with head and visual challenges.
  ○ Over-ground walking with horizontal/vertical head turns, variable speed, figure-of-eight paths, and navigation near visually busy areas (hallway posters, patterned floor).
5. Symptoms/self-regulation and education.
  ○ Brief breathing/rhythm-pacing strategies, reassurance about expected transient symptom provocation, and graded exposure principles to reduce avoidance.

Progression criteria. Exercise intensity and complexity were increased when patients completed a task with correct technique and only mild, transient symptom escalation (target 2–4/10) that resolved within the session. Regressions were applied if symptoms were sustained or technique deteriorated.

Home program and adherence. Patients received a daily home program (10–20 min/day) mirroring supervised tasks (VOR ×1; brief habituation exposures; simple balance drills). Adherence was tracked with a checklist, reviewed each session. Patients were instructed to avoid prolonged avoidance behaviors and to space home exercises to allow symptom settling.

Safety/withdrawal. Exercises were paused or modified for severe or prolonged symptom exacerbation, new neurologic signs, or intercurrent illness. No adverse events requiring withdrawal occurred during supervised sessions.

Therapist and setting. All sessions were delivered by a licensed physiotherapist trained in vestibular rehabilitation under the supervision of a neurotologist.

### 2.6. Sample Size and Study Design

This study was conceived as an exploratory, single-center observational investigation with a fixed recruitment window (June–August 2022). A formal a priori power analysis was not performed because effect-size estimates for VRT specifically in PPPD populations were limited at the time and the primary aim was feasibility and signal detection. We enrolled consecutive eligible patients during the study period. The sample was not powered to detect small between-subgroup effects (e.g., sex interactions); therefore, we report exact *p*-values and 95% confidence intervals to reflect statistical uncertainty.

### 2.7. Statistical Analysis

Descriptive statistics are reported as mean ± standard deviation (SD) for continuous variables and *n* (%) for categorical variables. Normality of continuous variables was assessed using the Shapiro–Wilk test. Longitudinal changes were analyzed using generalized estimating equations (GEE) with a normal distribution and identity link to account for within-subject correlations across time points (T1, T2, T3). The model included moment (time), sex, and their interaction (moment × sex). An exchangeable working correlation structure with robust (sandwich) standard errors was specified. Model parameters were evaluated with Wald tests. We report EMM ± SE for each time point and pairwise mean differences with 95% CI from the GEE model; exact two-sided *p*-values are provided. Statistical significance was set at *p* ≤ 0.05 (two-sided). Analyses were performed using IBM SPSS Statistics, Version 21.0 (IBM Corp., Armonk, NY, USA).

## 3. Results

This is, to our knowledge, the first report from a cohort recruited in Lithuania. We included 25 patients (mean age 44.48 ± 14.43 years); 21 (84%) were women. At baseline (T1), mean DHI scores were: Functional 16.81 ± 2.12, Emotional 11.49 ± 1.24, Physical 15.04 ± 1.26, and Total 43.33 ± 4.15 (Table 1).

Functional domain. EMM (95% CI): T1 16.81 ± 2.12 (12.65–20.96), T2 10.12 ± 1.87 (6.45–13.79), T3 9.70 ± 1.91 (5.49–13.46) (Table 2). Time effect *p* = 0.023; sex *p* = 0.360; time × sex *p* = 0.572. Pairwise differences: T2 − T1 Δ = −6.69 (*p* = 0.018, T3 − T1 Δ = −7.11; *p* = 0.013, T3 − T2 Δ = −0.42 (*p* = 0.876). Despite reductions from T1, overlapping CIs for T2 and T3 indicate weak evidence for additional change after T2.

Emotional domain. EMM (95% CI): T1 11.49 ± 1.24 (9.05–13.93), T2 7.37 ± 0.92 (5.55–9.18), T3 7.08 ± 0.96 (5.19–8.97) (Table 2). Time *p* = 0.010; sex *p* < 0.001; interaction *p* = 0.068. Women had higher (worse) emotional-domain scores than men (mean difference 5.63; SE 1.217; 95% CI 3.24–8.01; *p* < 0.001). Pairwise: T2 − T1 Δ = −4.12 (*p* = 0.008, T3 − T1 Δ = −4.40; *p* = 0.005, T3 − T2 Δ = −0.29; *p* = 0.831. Overlapping CIs between T2 and T3 suggest no clear incremental improvement beyond T2.

Physical domain. EMM (95% CI): T1 15.04 ± 1.26 (12.55–17.52), T2 11.26 ± 1.09 (9.11–13.42), T3 10.71 ± 1.07 (8.61–12.82) (Table 2). Time *p* = 0.022; sex *p* = 0.455; interaction *p* = 0.116. Pairwise: T2 − T1 Δ = −3.77 (*p* = 0.024, T3 − T1 Δ = −4.32 (*p* = 0.009, T3 − T2 Δ = −0.55 (*p* = 0.722. Overlapping CIs at T2 and T3 again indicate weak evidence for further change after T2.

DHI total. EMM (95% CI): T1 43.33 ± 4.15 (35.19–51.48), T2 28.75 ± 3.07 (22.72–34.78), T3 27.50 ± 3.40 (20.82–34.18) (Table 2). Time *p* = 0.006; sex *p* = 0.103; interaction *p* = 0.167. Pairwise: T2 − T1 Δ = −14.58 (*p* = 0.005, T3 − T1 Δ = −15.83 (*p* = 0.003, T3 − T2 Δ = −1.25 (*p* = 0.785. The cohort shifted from the moderate DHI range at baseline (43.33, 31–60) to the mild range at both T2 (28.75) and T3 (27.50). Reductions from T1 to T2 and from T1 to T3 exceeded the MCID ≈11–18 points, while the small T3−T2 change with overlapping CIs provides weak/absent evidence for further improvement beyond T2. Mean change scores and severity-range shifts are summarized in Table 3.

Overall, several time effects were statistically significant, but 95% CIs were relatively wide and often overlapping, so effect magnitudes should be interpreted with caution. Findings indicate a trend toward improvement after vestibular rehabilitation therapy, maintained at 3-month follow-up.

## 4. Discussion

Our study demonstrates improvements in disease-specific quality of life, as reflected by the DHI total score, during a supervised clinic/hospital-based VRT program, with gains maintained at three months. Given the impact of PPPD on daily functioning, a previous study reported higher DHI total scores and elevated depression and anxiety in individuals with PPPD compared with those with peripheral vestibular disorders such as BPPV, Ménière’s disease, or vestibular neuritis [9]. In our cohort, the largest improvements occurred from T1 → T2, while T2 → T3 showed no additional significant change in the functional, emotional, or physical domains, consistent with stabilization after active therapy. Time (moment) effects were significant, whereas sex and time × sex were not significant for functional, physical, or total scores; women had higher (worse) emotional-domain scores than men across time points. These findings are important for clinical practice, showing that a short, structured VRT program can lead to lasting improvements in daily functioning and emotional well-being. By reducing dizziness-related handicap and sustaining benefits beyond therapy, it provides a practical and accessible rehabilitation option for PPPD. Overall, the results highlight the importance of VRT as an effective first-line, non-pharmacological approach for personalized care.

Mechanistic interpretation (focus)

The observed pattern aligns with plausible PPPD mechanisms. First, sensory reweighting likely reduced visual dependence and improved vestibular–proprioceptive integration through graded exposure to upright posture, motion, and visually complex environments. Second, gaze-stabilization (VOR ×1/± ×2) exercises plausibly enhanced visual acuity during head movement, diminishing motion-provoked dizziness and functional limitations. Third, habituation to provocative visual motion may have attenuated threat anticipation and avoidance behaviors. The higher emotional scores in women suggest affective modulation of perceived handicap, supporting routine screening and targeted management of anxiety/depression alongside VRT [1,5,11]. Notably, DHI total reductions exceeded the MCID (~11–18 points), indicating clinically meaningful change; by contrast, small T2 → T3 differences with overlapping 95% CIs indicate weak/absent evidence for further gains beyond the supervised phase.

Relation to prior work (concise, non-redundant)

Our domain-level findings are consistent with reports of functional and physical improvements after ~6 weeks of VRT [16,17,18,19] and with clinically relevant DHI reductions in self-managed/home-based and hospital-based programs [14,15,16,18,19]. We deliberately interpret effects using exact *p*-values, 95% CIs, and DHI severity categories (mild 0–30, moderate 31–60, severe 61–100) to distinguish statistical from clinical significance. Our findings are concordant with recent aggregate evidence from systematic reviews/meta-analyses that report improvements in DHI with VRT, while emphasizing heterogeneity and the need for robust randomized designs [10].

Delivery mode and comparators

The current protocol was supervised, clinic/hospital-based VRT delivered twice weekly for five weeks by a licensed physiotherapist under neurotologist supervision, with a brief daily home program as an adjunct (not a self-managed-only protocol). Although prior reports indicate that both supervised/hospital-based and home-based programs can reduce DHI [15,16,17,18,19], our investigation did not include a home-based-only comparator; therefore, success-rate differences between modalities cannot be inferred. Future randomized studies should directly compare hospital-based versus home-based (or hybrid) VRT, with harmonized dosing and adherence monitoring.

Durability and expected trajectory. The largest gains occurred during supervised VRT (T1 → T2) and were maintained at 3 months (T3), suggesting a plateau after the primary adaptation phase. Whether these benefits persist, grow with continued practice (‘booster’ sessions), or attenuate without supervision remains unknown. Notably, PPPD-specific VRT trials with follow-up beyond 3 months are scarce, and systematic reviews have highlighted the paucity of outcomes reported at ≥6–12 months [10,11,12]. Accordingly, studies with longer follow-up, standardized home-program adherence tracking, and prespecified maintenance/booster strategies are needed to define long-term sustainability.

Limitations

This study has several limitations that should be acknowledged. First, as a prospective observational design without a control or comparator group, causal inference is limited, and the possibility of natural recovery or placebo effects cannot be excluded. The relatively small sample size (N = 25) and lack of an a priori power calculation reduce statistical precision and the ability to detect subgroup effects, resulting in wide confidence intervals; therefore, the findings should be considered exploratory. Follow-up was restricted to three months, preventing conclusions about long-term sustainability (≥6–12 months). Additionally, potential confounders such as daily physical activity levels, psychological comorbidities, and concurrent treatments were not fully controlled, which may have influenced the outcomes. Finally, the study was conducted in a single-center cohort in Lithuania; thus, generalizability to other populations or cultural contexts is limited. Given the observational design and absence of a comparator group, improvements observed cannot be attributed to the intervention with certainty, underscoring the need for future randomized controlled trials to confirm these findings.

## 5. Conclusions

In this single-center cohort with persistent postural-perceptual dizziness (PPPD), supervised clinic-based vestibular rehabilitation therapy (VRT)—complemented by a brief home program—produced statistically and clinically meaningful improvements in Dizziness Handicap Inventory (DHI) total and subdomain scores. Most patients improved from moderate to mild handicap, with benefits sustained at three months. Women reported higher emotional-domain scores, emphasizing the importance of addressing anxiety and depressive symptoms as part of comprehensive PPPD management. The observed pattern is consistent with key VRT mechanisms, including sensory reweighting, habituation, and improved gaze stabilization, which together reduce motion sensitivity and visual dependence. Clinically, these findings reinforce VRT as a practical and effective core component of PPPD care, capable of improving daily functioning and quality of life. The magnitude of improvement exceeded the minimal clinically important difference (MCID), supporting its real-world therapeutic relevance. Future randomized studies with longer follow-up are warranted to confirm efficacy, optimize delivery modes, and assess long-term sustainability of outcomes.

## Figures and Tables

**Table 1:** Baseline characteristics of the study population.

Characteristic	Value
Participants, *n*	25
Age, years	44.48 ± 14.43
Female, *n* (%)	21 (84%)
DHI—Functional subscore (0–36) at T1	16.81 ± 2.12
DHI—Emotional subscore (0–36) at T1	11.49 ± 1.24
DHI—Physical subscore (0–28) at T1	15.04 ± 1.26
DHI—Total score (0–100) at T1	43.33 ± 4.15

Notes. Values are mean ± SD unless stated otherwise; categorical data are *n* (%). T1 = baseline; T2 = post-intervention (week 5); T3 = 3-month follow-up without additional VRT.

**Table 2:** DHI outcomes across time (EMM ± SE; 95% CI) and pairwise *p*-values.

Metric	T1 (EMM ± SE; 95% CI)	T2 (EMM ± SE; 95% CI)	T3 (EMM ± SE; 95% CI)	*p* (T2 − T1)	*p* (T3 − T1)	*p* (T3 − T2)
DHI—Functional (0–36)	16.81 ± 2.12 (12.65–20.96)	10.12 ± 1.87 (6.45–13.79)	9.70 ± 1.91 (5.49–13.46)	0.018	0.013	0.876
DHI—Emotional (0–36)	11.49 ± 1.24 (9.05–13.93)	7.37 ± 0.92 (5.55–9.18)	7.08 ± 0.96 (5.19–8.97)	0.008	0.005	0.831
DHI—Physical (0–28)	15.04 ± 1.26 (12.55–17.52)	11.26 ± 1.09 (9.11–13.42)	10.71 ± 1.07 (8.61–12.82)	0.024	0.009	0.722
DHI—Total (0–100)	43.33 ± 4.15 (35.19–51.48)	28.75 ± 3.07 (22.72–34.78)	27.50 ± 3.40 (20.82–34.18)	0.005	0.003	0.785

Notes: Values are estimated marginal means (EMM) ± standard error (SE) with 95% confidence intervals (CI). Pairwise *p*-values are from generalized estimating equations (normal distribution, identity link, exchangeable correlation; Wald tests). T1 = baseline; T2 = post-intervention (week 5); T3 = 3-month follow-up without additional VRT.

**Table 3:** Mean changes (Δ) in DHI outcomes and clinical interpretation.

Metric	Δ T2 − T1 (95% CI)	Δ T3 − T1 (95% CI)	Δ T3 − T2 (95% CI)	Clinical Interpretation
DHI total (0–100)	−14.58 (—)	−15.83 (—)	−1.25 (—)	T1 → T2 and T1 → T3 exceed MCID; T3 → T2 not clinically meaningful
Functional (0–36)	−6.69 (—)	−7.11 (—)	−0.42 (—)	Improves during supervised phase; minimal additional change at follow-up
Emotional (0–36)	−4.12 (—)	−4.40 (—)	−0.29 (—)	Improves and maintained; women scored higher (worse) than men
Physical (0–28)	−3.77 (—)	−4.32 (—)	−0.55 (—)	Improves during supervised phase; maintained at follow-up

Notes. Δ values are arithmetic differences between time points (negative = improvement). Where available, report 95% CI from the GEE contrasts; cells are marked as “—” pending CI values. DHI Total severity ranges: mild 0–30, moderate 31–60, severe 61–100. MCID for DHI Total ≈ 11–18 points.

## Data Availability

The datasets generated and analyzed during the current study are available from the corresponding author on reasonable request.

## Abbreviations

PPPD	Persistent Postural-Perceptual Dizziness
VRT	vestibular rehabilitation therapy
CBT	cognitive behavioral therapy
RCT	randomized control trial
DHI	dizziness Handicap Inventory
HRQoL	physical health-related quality of life
GEE	Generalized Estimating Equations
